# Silicon Protects Rice Plants Against Striped Stem Borer by Disturbing Herbivory-Induced Putrescine Accumulation

**DOI:** 10.3390/plants14132066

**Published:** 2025-07-06

**Authors:** Hao Zhang, Xiaodong Liu, Cunyan Li, Linzhi Fang, Chaoyue Gai, Rensen Zeng, Qiongli Wang, Yuanyuan Song, Daoqian Chen

**Affiliations:** 1 Key Laboratory of Ministry of Education for Genetics, Breeding and Multiple Utilization of Crops, Key Laboratory of Ministry of Agriculture and Rural Affairs of Biological Breeding for Fujian and Taiwan Crops, College of Agriculture, Fujian Agriculture and Forestry University, Fuzhou 350002, China; 15864667335@163.com (H.Z.); 17352015063@163.com (X.L.); 15987282757@163.com (C.L.); amazingseed@126.com (L.F.); gaichaoyuekaixin@163.com (C.G.); rszeng@fafu.edu.cn (R.Z.); 2 Fujian Provincial University Key Laboratory of Crop Biotechnology, Fujian Agriculture and Forestry University, Fuzhou 350002, China

**Keywords:** Silicon (Si), *Oryza sativa*, striped stem borer, polyamines, anti-herbivore resistance

## Abstract

Silicon (Si) protects plants against insect herbivores; however, the underlying mechanisms remain unclear. Polyamines (PAs) play a crucial role in plant–insect interactions. Here, the involvement of Si in putrescine (Put) metabolism and its role in rice resistance against striped stem borer (SSB, *Chilo suppressalis* Walker) were investigated. The results showed that SSB larval infestation led to a substantial accumulation of free Put in rice seedlings. Si application increased rice resistance against SSB and repressed the SSB attack-induced accumulation of Put, in parallel with a decreased expression of Put biosynthesis genes encoding arginine decarboxylase (*ADC1* and *ADC2*). Moreover, Si application had no significant effect on the wounding-induced expression of *ADC1* and *ADC2*, but attenuated the further elevation in the transcription of *ADC1* and *ADC2* induced by SSB larvae oral secretion. Simultaneously, Si addition reduced the Put and spermidine contents in SSB-attacked plants. Furthermore, the exogenous application of Put attenuated Si-enhanced resistance against SSB larvae, whereas exogenous D-arginine, an inhibitor of ADC, showed similar effects to Si on rice resistance against SSB. Our findings indicate that Si improves rice resistance to SSB, at least partly by reducing herbivory-stimulated putrescine accumulation.

## 1. Introduction

Rice (*Oryza sativa* L.) is the staple food for half the population of the world, and it is seriously impacted by insect pests that threaten its global productivity and food security. [1]. Rice striped stem borer (SSB, *Chilo suppressalis* Walker) is a notorious pest in rice-growing regions worldwide [2]. SSBs attack by boring into the culm and feeding inside, resulting in “dead hearts” with desiccated main culms at the tillering stage and a “whitehead” with withered spikes at the reproduction stage, leading to yield losses in rice production [3,4]. The boring behavior and overlapping generations of SSBs, as well as the absence of SSB resistance genes in the rice germplasm, have always imposed a challenge for controlling this notorious pest. To date, the mainstay for crop protection against SSBs has been chemical insecticides. The excessive use of chemical pesticides has caused increasing insect resistance to pesticides, environmental toxicity, and concerns for human health [5]. Hence, it is critical to develop novel sustainable strategies to control SSBs.

Although Si is not recognized as an essential element for the majority of plants, its beneficial roles in enhancing plant tolerance to various environmental stresses have been well documented in a number of plant species, especially in rice, which accumulates high levels of Si (up to 10% of total plant dry mass) [6,7]. Numerous studies have also reported that Si application could protect rice plants against insect herbivores, including SSBs [8,9,10,11]. The physical barrier formed by Si deposition has long been considered a major cause of Si-enhanced plant resistance to insect pests. Si deposited in plant tissues increases their rigidity and abrasiveness, thereby decreasing insect damage [12]. Recent studies also identified that Si could play an active role in protecting plants against insect herbivores by enhancing direct and indirect plant defenses, as well as defense priming [13]. Moreover, it is proposed that apoplastic Si deposition might function as an apoplastic obstruction that interferes with effector release, translocation, and/or host recognition under pathogenic fungal or pest attack [14,15], but the experimental evidence supporting this hypothesis remains scarce.

Polyamines (PAs) are ubiquitous low-molecular-weight aliphatic amines that exist across all living organisms and are involved in signal transduction, transcriptional regulation, ion channel function, DNA and protein synthesis, etc. [16,17]. Putrescine (Put), spermidine (Spd), and spermine (Spm) are the most common PAs in plants [18]. Put is synthesized from arginine and ornithine via the catalytic actions of arginine decarboxylase (ADC) and ornithine decarboxylase (ODC), respectively. PAs’ biosynthetic pathway in plants, which can generate Put from both L-arginine and L-ornithine, differs from that in animals, where L-ornithine is the sole precursor. Then, Put subsequently receives an aminopropyl moiety via the catalytic actions of spermidine synthase (SPDS) to produce Spd, which is further transformed into Spm by spermine synthase (SPMS). The aminopropyl moieties required for these reactions are donated by decarboxylated S-adenosylmethionine (dcSAM), a compound synthesized by S-adenosyl-methionine decarboxylase (SAMDC) using S-adenosyl-methionine (SAM) as a substrate [19].

The involvement of PAs in plant disease resistance has been widely reported. The induction of PA biosynthesis and oxidation by pathogen attack is recognized to be important for plant defense through triggering the hypersensitive response [20,21,22]. An increase in plant PA biosynthesis is also observed in several plant–pest interactions [23,24,25,26]. The increased PAs are proposed to function in plant defense as phenolamides, which could block the glutamatergic neuromuscular junctions and lead to the paralysis of insects’ skeletal muscles [23,24]. However, contrasting evidence has indicated that pathogenic Hessian fly (*Mayetiola destructor*) larvae stimulate wheat PA biosynthesis to acquire their own required nutrient substances [25]. A triticale cultivar susceptible to bird cherry-oat aphid (*Rhopalosiphum padi* L.) was characterized by a higher content of Put and Spm compared to a less susceptible triticale cultivar [26]. Recently, it was revealed that the exogenous application of Spd enhances brown planthopper (BPH) feeding, and *pao6* mutants with higher levels of Spd showed decreased resistance to BPH compared to wild-type rice plants [27]. Thus, the function of the elicitation of PA biosynthesis in the host plant apparently varies in different pest–plant interactions. Our previous study on rice–SSB interaction reveals that SSB larval infestation triggers Put accumulation in host rice plants, which facilitates SSB growth [28].

Previous studies have indicated that PAs are involved in Si-mediated resistance to salt toxicity, drought stress, potassium deficiency, and gummosis [29,30,31,32,33,34]. Here, the potential involvement of Si-mediated Put metabolism in Si-enhanced rice resistance to SSB and its underlying mechanisms were investigated. Our results demonstrated that Si improves rice resistance to SSB, at least partly by repressing the SSB attack-increased accumulation of Put. Si application attenuated the elicitation of Put biosynthesis by SSB larval oral secretion, but not wounding alone. This study provides evidence that Si plays a role in interfering with the metabolic manipulation of host plants by insect herbivores rather than acting as a mere mechanical barrier or defense activator during plant–pest interactions.

## 2. Results

### 2.1. Effects of Si on Rice Resistance to SSB

**Figure 1:**
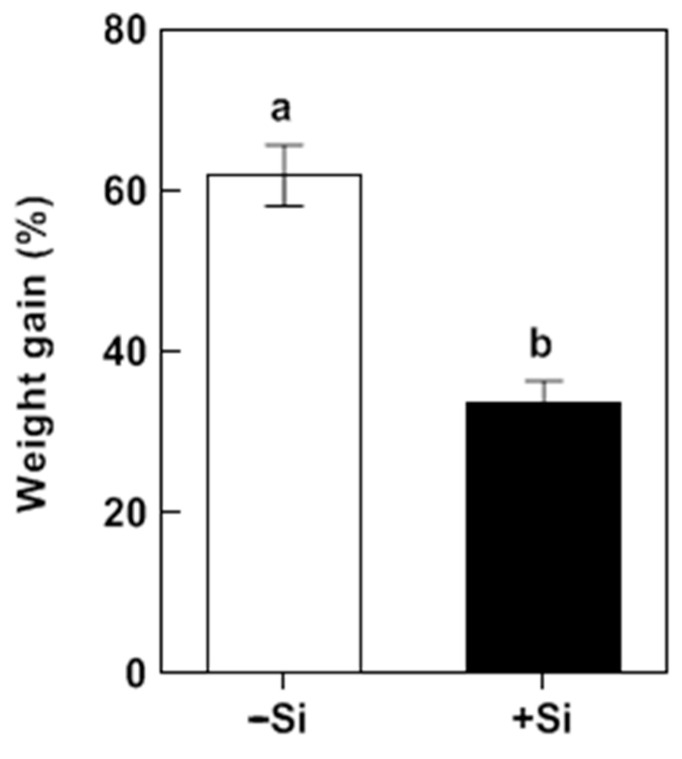
Effects of Si on weight gain of *Chilo suppressalis* (SSB) larvae fed on rice plants. Values are mean ± SE (*n* = 20). Letters above bars indicate significant differences among treatments (*p* < 0.05 according to Student’s *t* test).

In the Si-treated plants, the shoot Si content increased by 5.75-fold relative to plants without Si treatment (Figure S1). Si application significantly decreased the SSB larval weight gain during 3 d of infestation. SSB larvae fed on plants without Si application increased in mass by 61.9% 3 d after infestation, whereas larvae fed on Si-treated plants increased in mass by only 33.8% (Figure 1).

### 2.2. Effects of Si on SSB Attack-Induced Put Accumulation in Rice

**Figure 2:**
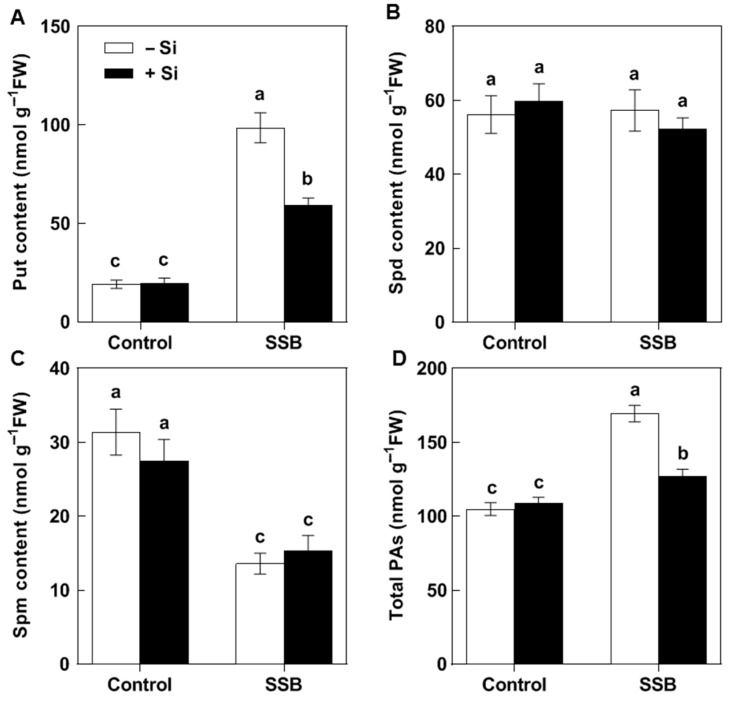
The effects of Si on polyamine levels in the rice plants attacked by *Chilo suppressalis* (SSB). Putrescine (Put, **A**), spermidine (Spd, **B**), spermine (Spm, **C**), and total polyamines (**D**) content in SSB-attacked rice plants. The values are mean ± SE (*n* = 4). The letters above the bars indicate significant differences among the treatments (*p* < 0.05 according to Tukey’s multiple range test).

Si treatment by itself did not significantly alter the levels of all tested PAs (Figure 2). SSB attack resulted in substantial increases in Put and total PA levels and a decrease in Spm levels in plants without Si treatment, while Spd levels remained unchanged after SSB attack (Figure 2A–D). Si treatment significantly reduced the SSB attack-induced Put accumulation, but had no effect on the Spd and Spm levels (Figure 2A–C). In plants without Si treatment, Put levels increased by 5.13-fold relative to plants not exposed to SSBs, whereas Put levels increased by only 2.99-fold in the Si-treated plants (Figure 2A). These results indicate that Si application decreased the substantial SSB attack-induced accumulation of Put and that this might be partly responsible for Si’s beneficial effect on rice resistance to SSBs.

### 2.3. Effects of Si on SSB Attack-Induced Expression of Rice Put Biosynthesis Genes

**Figure 3:**
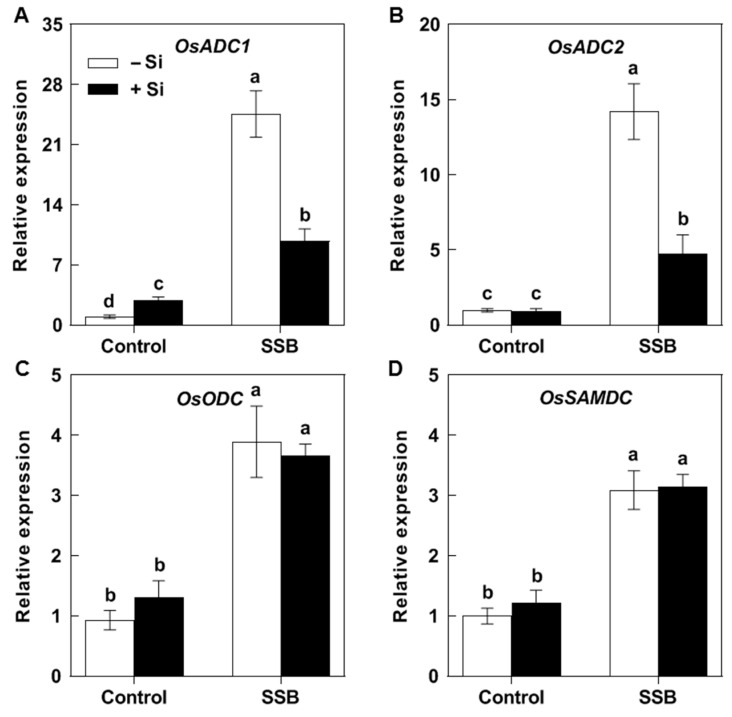
The effect of Si on the expression of polyamine biosynthesis genes in the rice plants attacked by *Chilo suppressalis* (SSB). The transcript levels of genes encoding arginine decarboxylase (*OsADC1*, **A**; *OsADC2*, **B**), ornithine decarboxylase (*OsODC*, **C**), and S-adenosylmethionine decarboxylase (*OsSAMDC*, **D**) in SSB-attacked rice plants. The values are mean ± SE (*n* = 3). The letters above the bars indicate significant differences among the treatments (*p* < 0.05 according to Tukey’s multiple range test).

In the absence of SSB damage, Si supplementation slightly increased the expression of *OsADC1* (Figure 3A), but had no significant effect on the transcript levels of *OsADC2*, *OsODC*, or *OsSAMDC* (Figure 3B–D). The mRNA levels of all four tested genes were increased in both Si-treated and untreated seedlings after SSB attack, but the magnitude of induction of *OsADC1* and *OsADC2* was lower in the Si-treated seedlings (Figure 3A,B). Si supplementation had no significant effect on the transcript levels of *OsODC* and *OsSAMDC* after SSB attack (Figure 3C,D). In the plants without Si treatment, the transcript levels of *OsADC1* and *OsADC2* increased by 23.64- and 13.21-fold, respectively, relative to the plants not exposed to SSB, whereas in the Si-treated plants, they increased by only 6.93- and 3.78-fold, respectively (Figure 3A,B).

### 2.4. The Effects of Si on the Induction of Put Biosynthesis Genes by SSB Larval Oral Secretion

**Figure 4:**
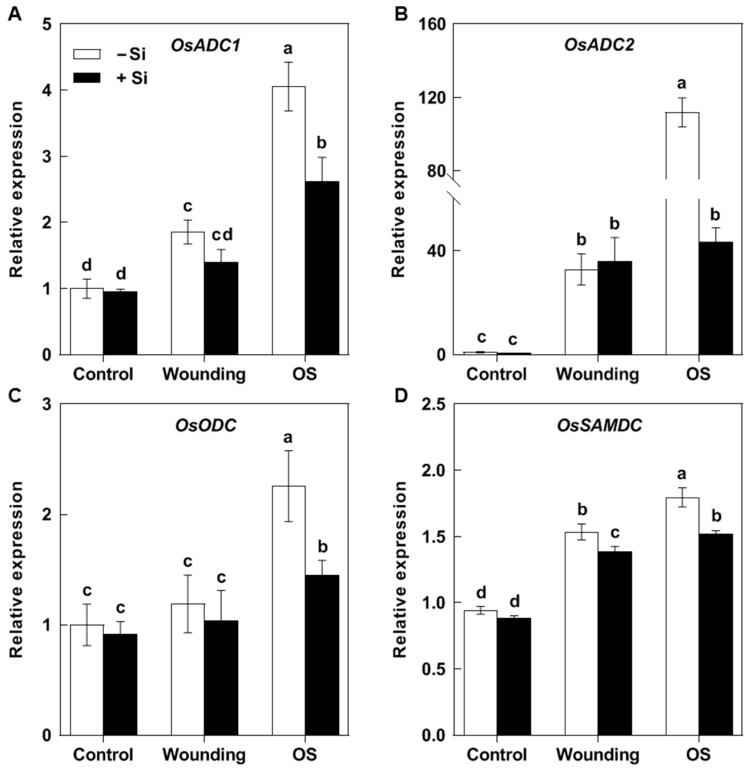
Effect of Si on expression of polyamine biosynthesis genes in wounded rice plants treated with *Chilo suppressalis* larvae oral secretion (OS). Transcript levels of genes encoding arginine decarboxylase (*OsADC1*, **A**; *OsADC2*, **B**), ornithine decarboxylase (*OsODC*, **C**), and S-adenosylmethionine decarboxylase (*OsSAMDC*, **D**) in SSB oral secretion-treated rice plants. Values are mean ± SE (*n* = 3). Letters above bars indicate significant differences among treatments (*p* < 0.05 according to Tukey’s multiple range test).

In the plants without Si supplementation, the transcript levels of all tested PA biosynthesis genes were increased by both mechanical wounding and OS treatment, but the magnitude of induction was significantly higher in the OS-treated plants. Si application depressed the further OS-mediated elevation but not the wounding-mediated primary induction of the transcription of all tested PA biosynthesis genes, especially for *OsADC2*. In plants without Si supplementation, the transcript levels of *OsADC2* were increased by 31.79- and 110.86-fold by wounding and OS treatment, respectively, whereas in Si-treated plants, they increased by 35.37- and 42.66-fold, respectively (Figure 4B). These results suggest that Si application might function by interfering with the SSB effector-based manipulation of host Put biosynthesis.

### 2.5. The Effects of Si on PA Levels in the SSB Larvae Fed on the Rice Plants

**Figure 5:**
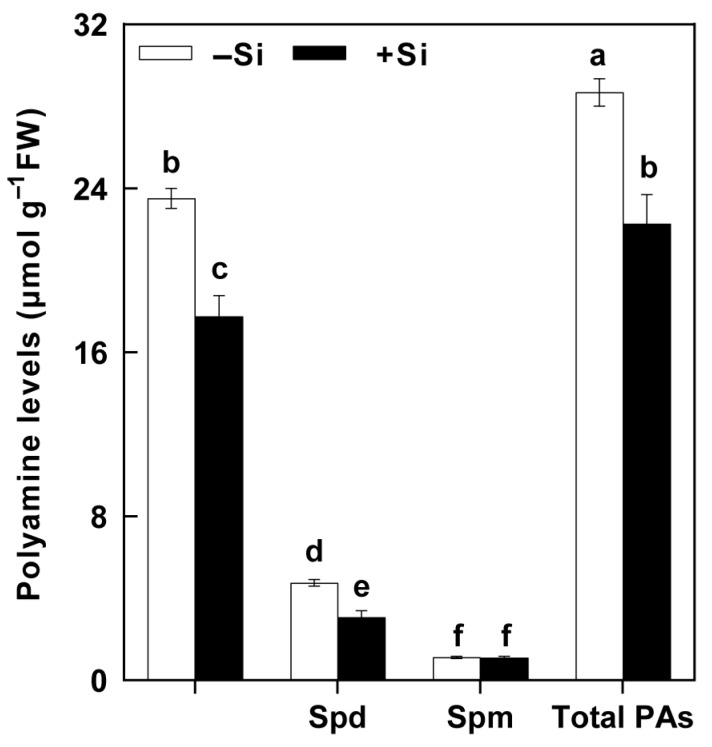
Polyamine levels in *Chilo suppressalis* larvae fed on Si-treated and untreated rice plants. Putrescine (Put), spermidine (Spd), spermine (Spm), and total polyamine content in SSB larvae fed on rice plants. Values are mean ± SE (*n* = 4). Letters above bars indicate significant differences among treatments (*p* < 0.05 according to Tukey’s multiple range test).

Si treatment significantly reduced the Put and Spd levels in SSB larvae fed on rice plants, but had no effect on the Spm level. The Put and Spd level in SSB larvae fed on Si-treated rice plants decreased by 20% and 20%, respectively, relative to those fed on untreated plants (Figure 5). These results indicate that Si application to rice plants could reduce the accumulation of PAs in SSB larvae fed on them.

### 2.6. Effects of Exogenous Application of Put on Si-Enhanced Resistance to SSB

Similarly to the beneficial effect of Si on rice resistance, the application of D-Arginine, an inhibitor of plant Put synthesis, to the rice plants also significantly decreased the weight gain rates of SSB larvae fed on them. In contrast, the protective role of Si for rice against SSB almost disappeared. These results indicated that Si-inhibited Put accumulation during rice–SSB interaction is critical for Si-enhanced rice resistance to SSB.

## 3. Discussion

**Figure 6:**
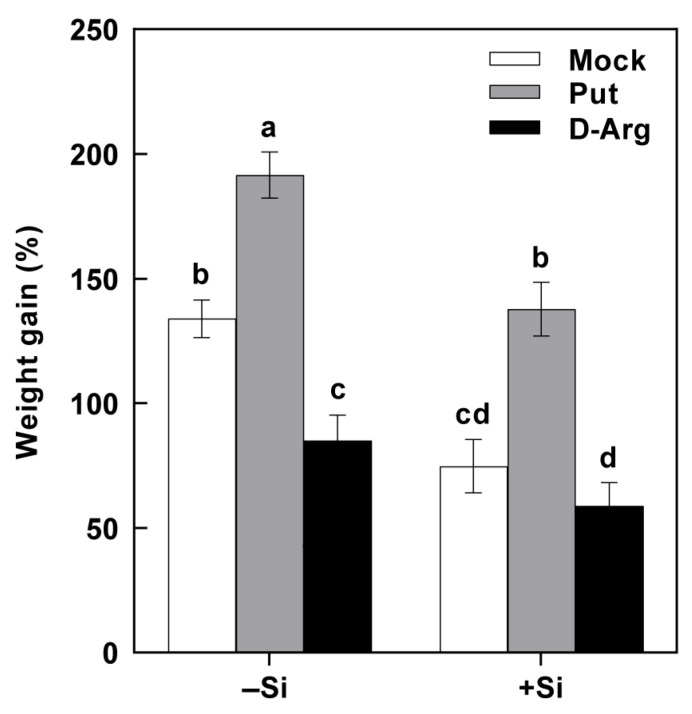
The effects of putrescine and D-arginine on the weight gain of *Chilo suppressalis* (SSB) larvae fed on rice plants treated with or without Si. Three-week-old seedlings supplied or not supplied with 2 mM Na_2_SiO_3_ were treated with putrescine (Put, 0.5 mM) and D-arginine (D-Arg, an inhibitor of arginine decarboxylase, 1 mM). The values are mean ± SE (*n* = 20). The letters above the bars indicate significant differences among treatments (*p* < 0.05 according to Tukey’s multiple range test).

Rice is a major food crop that absorbs and accumulates abundant Si in tissues, and it is recognized as a typical Si accumulator [35,36]. The abundant Si accumulated in rice plants is considered to play a role in protecting rice against various environmental stresses, including insect herbivores [8,37]. However, the underlying mechanisms of such protection remain elusive. In the present study, we found that Si significantly increases rice resistance to SSB, a notorious pest for worldwide rice production (Figure 1). Si application repressed the substantial SSB attack-induced accumulation of Put in rice plants, as well as the PA levels in SSB larvae fed on rice plants, associated with decreased transcripts of rice Put biosynthesis genes (Figure 2, Figure 3 and Figure 5). Furthermore, the exogenous application of Put attenuated Si-enhanced resistance against SSB larvae, whereas exogenous D-arginine (an inhibitor of Put synthesis) mimicked silicon’s effects on enhancing rice resistance to SSB larvae (Figure 6). These results collectively indicate that Si-mediated PA metabolism is critical for Si-enhanced resistance to insect herbivores. In addition, our results also suggest that SSB attack-induced rice Put biosynthesis might be caused by metabolic manipulation by SSB larvae in an effector-based manner, and Si application could effectively obstruct this progress (Figure 4). Therefore, this study provides evidence that Si enhances rice resistance to insect herbivores through mediating important metabolism processes rather than acting as a mere mechanical barrier or defense activator during plant–pest interaction.

PAs are aliphatic amines with a low molecular weight that are ubiquitously found in all living organisms, including plants, animals, and bacteria [38,39]. PAs are crucial regulators of growth, development, and stress responses in plants, while they are considered vital nutritional compounds for normal development in insect herbivores [40,41,42]. The involvement of PAs in plant–insect interactions has been reported in several studies. It has been found that pathogenic Hessian fly (*Mayetiola destructor*) larvae could stimulate wheat PA biosynthesis to acquire their own required nutrient substances [25]. A triticale cultivar susceptible to bird cherry-oat aphid (*Rhopalosiphum padi* L.) was associated with higher contents of Put and Spm compared to a less susceptible triticale cultivar [26]. A recent study on rice–BPH interactions suggests that BPH-stimulated Spd levels may contribute to modulating herbivore feeding activity [27]. Our recent study on rice–SSB interactions found that SSB larvae could also trigger Put accumulation in host rice plants to benefit their own growth [28]. In the present study, our results indicated that Si supplementation depressed the SSB-induced accumulation of Put in rice plants, as well as the Put and Spd levels in SSB larvae fed on rice plants, which were correlated with decreased transcripts of *OsADC1* and *OsADC2* (Figure 2, Figure 3 and Figure 5). Moreover, the results of the exogenous application of Put and Put biosynthesis inhibitor further confirmed that Si enhances rice resistance to SSB, at least partly by depressing SSB-induced Put accumulation. Similarly, the beneficial role of Si in enhancing plant resistance to stress by mediating PA metabolism was also observed in plant responses to both biotic and abiotic stresses, including salt toxicity, drought stress, potassium deficiency, and gummosis [29,30,31,32]. These results suggest that the regulatory role of Si on plant PA metabolism seems to extensively exist across plant responses to various environmental stresses.

Although there is no doubt regarding the beneficial role of Si in protecting plants against stress, both biotic and abiotic, the underlying mechanisms of such protection remain elusive, and the information available on how Si mediates metabolic pathways in plants is still limited [7,43,44,45]. The secretion of a repertoire of effectors from salivary glands into host cells to subvert plant immunity and facilitate insect feeding is recognized as an important counter-defense strategy of insect herbivores [46]. Obligate plant feeders generally employ resource manipulation in the host plant via effector-based mechanisms to ensure their sustenance [47,48]. In our previous study, we found that SSB attack-induced rice Put biosynthesis might be caused by metabolic manipulation by SSB larvae in an effector-based manner. Recently, it was proposed that effectors from pathogens or insects might be trapped within the extracellular Si matrix, obstructing their manipulation of plant immunity [15]. In this study, Si application had no significant effect on the wounding-induced expression of *ADC1* and *ADC2*, but it attenuated the further elevation of the transcription of these Put biosynthesis genes by SSB larvae oral secretion. Therefore, it is possible that apoplastic-deposited Si might interfere with the function of effectors from SSB, thereby obstructing the manipulation of host rice Put biosynthesis and protecting the rice plants against SSB attack.

Taken together, our findings indicate that the protection of Si in rice’s resistance to SSB is mediated at least partly by interfering with the SSB attack-induced Put biosynthesis. Our findings provide new insights into the function of Si in obstructing the insect herbivore effector-based manipulation of host metabolism. Further efforts towards elucidating the potential insect effectors manipulating host PA metabolism and clarifying the interactions between Si and insect effectors will provide more direct guidance for improving rice resistance against SSB.

## 4. Materials and Methods

### 4.1. Plant Materials and Growth Conditions

Rice (*Oryza sativa* L. cv. Nipponbare) seeds were surface-sterilized by soaking them in a 1.5% sodium hypochlorite solution for 15 min and then washing with distilled water 4 times. The sterilized seeds were sown in a seeding tray with wet gauze for germination. The 7-day-old seedlings were transplanted into a plastic box containing 5 L of full-strength modified Kimura B nutrient solution without Si (pH: 5.6, renewed every 3 d), as described by Zhang et al. [26]. The rice plants were cultivated in a growth chamber under a 12 h/12 h day/night cycle, with the temperature regime at 27 °C/23 °C, and a light intensity of 300 μmol m^−2^ s^−1^ for 2 weeks. After 2 weeks of cultivation, 80 uniform seedlings were selected and equally divided into two groups: +Si (full-strength modified Kimura B nutrient solution with 2 mM Na_2_SiO_3_·9H_2_O) and −Si (full-strength modified Kimura B nutrient solution with 0 mM Na_2_SiO_3_·9H_2_O). After 4 d of Si treatment, 20 plants from each group were subjected to herbivore infestation.

### 4.2. SSB Rearing and Infestation

The original population of the laboratory-maintained striped stem borer (*Chilo suppressalis* Walker) was generously provided by Professor Yunhe Li from the Institute of Plant Protection, Chinese Academy of Agricultural Sciences. SSB larvae were reared on artificial diets in an insectary with a 12h/12 h day/night cycle, temperature regime at 27 °C/25 °C, and relative humidity at 70–80% following Xue et al. [31]. The SSB colony had been maintained in the laboratory for over 20 generations before being used for our experiment. For herbivore infestation, twenty uniform third-instar SSB larvae (~20 mg) were weighed and recorded one by one, then caged on the rice plants (one larva per plant) with plastic tubes (SSB treatment). The control plants were fixed with empty plastic tubes. After 3 days, each larva was weighed and the weight gain percentage was calculated as the percentage of larvae weight gain after 3 d of feeding relative to the initial weight before infestation. The plant tissues around the feeding sites were sampled for further analysis.

### 4.3. Polyamine Quantification

The PA contents were determined using high-performance liquid chromatography (HPLC: LC-10A, Shimadzu, Kyoto, Japan) after extraction with 5% perchloric acid solution and derivatization with benzoyl chloride according to Zhang et al. [26]. 1,6-diaminohexane was used as an internal standard. Each treatment included four replicates.

### 4.4. Gene Expression Analysis

For gene expression analysis, frozen root samples (approximately 0.1 g) were used for RNA extraction. The gene expression analysis was conducted using a quantitative RT-PCR on the QuantStudio™ 1 Plus Real-Time PCR System (Applied Biosystems, Waltham, MA, USA), as previously described by Zhang et al. [26]. *OsActin* was used as an internal reference gene and gene-specific primers were the same as Zhang et al. [26]. The 2^−∆∆Ct^ method was used for the relative expression calculation. Each treatment included three biological replicates and each biological replicate included three technical replicates.

### 4.5. Oral Secretion Treatment

SSB oral secretions (OSs) were collected from third-instar larvae and diluted with sterile distilled water at a 1:9 (*v/v*) ratio according to Xue et al. [31]. Briefly, the larvae were gently stimulated on the mouthpart using a sterile micro-pipette to induce the regurgitation of OS; then, the droplets from the mouthpart were immediately collected and stored at −80 °C. After 4 d of Si treatment, the rice seedlings were mechanically wounded with a puncher at the main stem with identical spacing of 0.5 cm. Then, the wounded seedlings were supplied with 20 µL of diluted OS (OS treatment) or sterile water (wounding treatment) at the wounded sites. And the unwounded plants were used as a control (Control). After 1 h, the plant tissues around the wounded sites were sampled for further gene expression analysis as described above.

### 4.6. Exogenous Application of Put and D-Arginine

Three-week-old rice seedlings were subjected to three different treatments by applying Put or D-arginine in the nutrient solution with or without Si supplement (+Si, 2 mM Na_2_SiO_3_·9H_2_O; −Si, 0 mM Na_2_SiO_3_·9H_2_O): Mock (blank group without any treatment), Put (0.5 mM), and D-Arg (D-arginine, an inhibitor of arginine decarboxylase, 1 mM). Put and D-arginine were prepared as 50 × stock solutions in deionized water, then added proportionally to full-strength modified Kimura B nutrient solution. After 4 days of treatment, the plants were exposed to SSB larval infestation as described above.

### 4.7. Statistical Analysis

Statistical analysis was performed using the SPSS statistics software (Version 19.0 for Windows, SPSS, Chicago, IL, USA). The data were subjected to Student’s *t* test or analysis of variance (ANOVA) with Tukey’s test after Shapiro–Wilk test for normality, as well as Levene’s test for homoscedasticity for differences among treatments, and a *p* value ≤ 0.05 was considered significant between treatments. For the percentage data in Figure 1 and Figure 6, they were transformed by Arcsine transformation. All values are presented as the mean ± SE. Graphs were generated using GraphPad Prism 8.0 (GraphPad Software Inc, Boston, MA, USA).

## Data Availability

Data are contained within the article.

## Supplementary Materials

The following supporting information can be downloaded at: https://www.mdpi.com/article/10.3390/plants14132066/s1, Figure S1: Effects of Si on shoot Si accumulation.

## Abbreviations

The following abbreviations are used in this manuscript:

PAs	Polyamines
SSB	Striped stem borer
Si	Silicon
Put	Putrescine
Spd	Spermidine
Spm	Spermine
ADC	Arginine decarboxylase
ODC	Ornithine decarboxylase
SAMDC	S-adenosyl-methionine decarboxylase
OS	Oral secretion
D-Arg	D-arginine
HPLC	High-performance liquid chromatography
