# Supplementary material for: Silicon Protects Rice Plants Against Striped Stem Borer by Disturbing Herbivory-Induced Putrescine Accumulation
Source: Plants (Basel). 2025 Jul 6;14(13):2066. doi: 10.3390/plants14132066 (PMC12252242; doi:10.3390/plants14132066)
Supplement: Supplementary file 1 [file plants-14-02066-s001.zip › plants-3663449-supplementary.pdf]

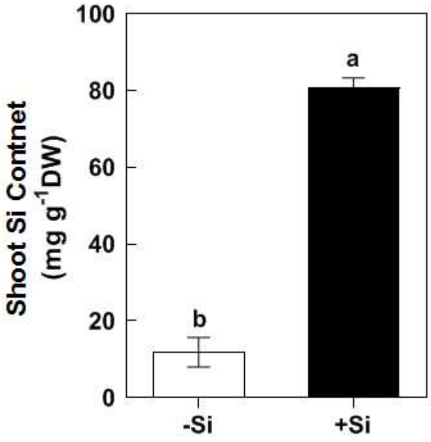

Figure S1. Effects of Si on shoot Si accumulation. Values are mean ± SE (n = 3). Letters above bars indicate significant differences among treatments ($p < 0.05$ according to Student t test).
